# Time Use, Gender, and Mental Health of Older Adults in Rural China

**DOI:** 10.1093/geroni/igab046.3128

**Published:** 2021-12-17

**Authors:** Zhiyong Lin, Feinian Chen

**Affiliations:** 1 The University of Texas at Austin, Austin, Texas, United States; 2 University of Maryland College Park, University of Maryland, Maryland, United States

## Abstract

Time use is considered a valuable descriptor of people’s lifestyles, and studying how people spend their time is critical for understanding the determinants and consequences of individual well-being. In this study, we first develop a time use typology to characterize how older adults in rural Chinese families allocate their time in later life, and then examined how older adults’ time allocation influenced their mental health, with a special focus on differential implications for older women and men. Data derived from 2015 and 2018 waves of a longitudinal study of 1,007 older adults, aged 60 and older, living in rural areas of Anhui Province, China. We specifically focused on how social and solitary dimensions of time use, as well as time spent within and outside households, impacted depressive symptoms of older adults. Using the K-means cluster analysis, we identified four time use categories: “work-oriented,” “socially-active,” “homemaker/ caretaker,” and “socially-isolated.” Results from fixed-effects regression analysis demonstrated that older women involved in “socially-active” time-use category tended to report better mental than those in other time-use types, while the time spent on housework and caregiving was harmful to their mental health. For older men, more time spent on paid activities outside households (“work-oriented”) was associated with better psychological outcomes while solitary leisure time (“socially-isolated”) was associated with higher levels of depressive symptoms. These findings will be helpful for health policymakers and practitioners who seek to better identify vulnerable subpopulations and to design effective intervention strategies to reduce mental health problems.

